# Comprehensive Overview on Multiple Strategies Fighting COVID-19

**DOI:** 10.3390/ijerph17165813

**Published:** 2020-08-11

**Authors:** Shaden A. M. Khalifa, Briksam S. Mohamed, Mohamed H. Elashal, Ming Du, Zhiming Guo, Chao Zhao, Syed Ghulam Musharraf, Mohammad H. Boskabady, Haged H. R. El-Seedi, Thomas Efferth, Hesham R. El-Seedi

**Affiliations:** 1 Department of Molecular Biosciences, The Wenner-Gren Institute, Stockholm University, S-106 91 Stockholm, Sweden; 2 Botany and Microbiology Department, Faculty of Science, Menoufia University, Menoufia 32511, Egypt; briksamsalah@gmail.com; 3 Department of Chemistry, Faculty of Science, Menoufia University, Menoufia 32511, Egypt; m_h_elashal@yahoo.com; 4 School of Food Science and Technology, National Engineering Research Center of Seafood, Dalian Polytechnic University, Dalian 116024, China; duming@dlpu.edu.cn; 5 School of Food and Biological Engineering, Jiangsu University, Zhenjiang 212013, China; guozhiming@ujs.edu.cn; 6 College of Food Science, Fujian Agriculture and Forestry University, Fuzhou 350002, China; zhchao@live.cn; 7 H.E.J. Research Institute of Chemistry, International Center for Chemical and Biological Sciences, University of Karachi, Karachi 75270, Pakistan; musharraf1977@yahoo.com; 8 Department of Physiology, School of Medicine, Mashhad University of Medical Sciences, Mashhad 9177948564, Iran; boskabadymh@mums.ac.ir; 9 Faculty of Medicine, Riga Stradins University (RSU), 16 Dzirciema iela, LV-1007 Riga, Latvia; haged.hr@gmail.com; 10 Department of Pharmaceutical Biology, Institute of Pharmaceutical and Biomedical Sciences, Johannes Gutenberg University, Staudinger Weg 5, 55128 Mainz, Germany; efferth@uni-mainz.de; 11 International Research Center for Food Nutrition and Safety, Jiangsu University, Zhenjiang 212013, China

**Keywords:** health care systems, global health responses, symptoms, strategies, economic recession

## Abstract

Lately, myriad of novel viruses have emerged causing epidemics such as SARS, MERS, and SARS-CoV-2, leading to high mortality rates worldwide. Thus, these viruses represented a challenging threat to mankind, especially considering the miniscule data available at our disposal regarding these novel viruses. The entire world established coordinative relations in research projects regarding drug and vaccine development on the external range, whereas on the internal range, all countries declared it an emergency case through imposing different restrictions related to their border control, large gatherings, school attendance, and most social activities. Pandemic combating plans prioritized all sectors including normal people, medical staff politicians, and scientists collectively shouldered the burden. Through planning and learning the previous lessons from SARS and MERS, healthcare systems could succeed in combating the viral spread and implications of these new pandemics. Different management strategies including social distance, social awareness and isolation represented successful ways to slow down the spread of the pandemic. Furthermore, pre-preparedness of some countries for emergencies is crucial to minimize the consequences of the crisis.

## 1. Introduction

Infectious diseases of new emerging pathogens or recurrent known ones lead to deaths among both human and animal populations. Infections can be transferred directly from human to human by respiratory droplets (i.e., measles), via flies (i.e., trypanosomiasis), or mosquitos (i.e., malaria), body secretions (i.e., chlamydia), or contamination via food or water (i.e., cholera) [1]. Some of the most devastating infectious diseases are brought about by viruses, including influenza, measles, and West Nile fever; furthermore, bacteria cause anthrax, salmonellosis, chlamydia, and cholera, and protozoa can cause malaria and trypanosomiasis.

**Figure 1:**
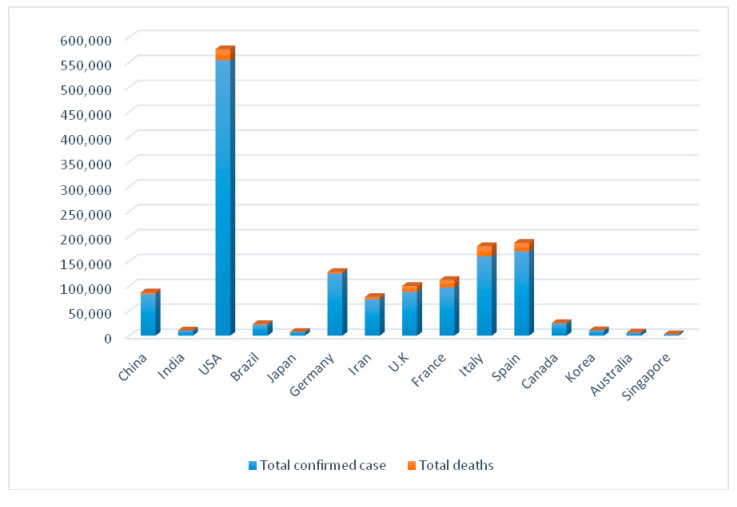
Comparison between total deaths and confirmed cases in some countries from 21 January till 14 April 2020 [10].

**Figure 2:**
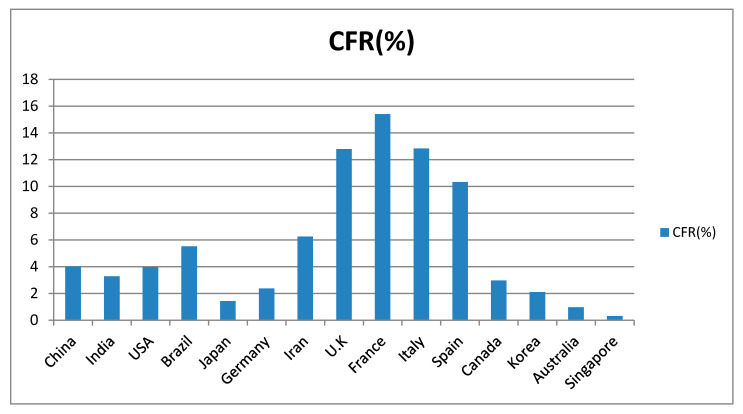
Case fatality rate (CFR) in some affected countries from 21 January till 14 April 2020 [10].

Coronaviruses (CoVs) are serious pathogens to humans and vertebrates affecting the hepatic, gastrointestinal, respiratory, and central nervous systems [2]. Coronaviruses belong to the Coronaviridae subfamily of the Nidovirales family. This subfamily is comprised of four genera: α-coronaviruses, β-coronaviruses, γ-coronaviruses, and δ-coronaviruses [3]. Various CoVs exhibit broad host ranges and tropism of tissue. Mammals typically get infected by α- and β-coronaviruses, whereas γ- and δ-coronaviruses typically affect birds and fish but only occasionally mammals [4]. The world has witnessed a series of coronavirus waves during the past two decades, triggering pandemics that have their perturbations on global health. The first one, named severe acute respiratory syndrome (SARS-CoV), had its last outbreak reported in September 2003 after infecting more than 8000 persons and causing 774 deaths with a fatality rate of 9.5 percent [5]. Nine years later, Middle East Respiratory Syndrome (MERS-CoV) triggered a respiratory disease in the Middle East. Even so; MERS-CoV is still ongoing compared to SARS-CoV, and the fatality rate was much higher (about 35%). Since December 2019 a third one called COVID-19 has emerged in Wuhan, Hubei province in China. COVID-19 is widely dispersed in humans and its genome is detached from SARS-CoV and MERS-CoV. China classified COVID-19 as a B-infectious disease according to the surveillance system of infectious diseases, which was updated in 2004, and categorized infectious diseases into classes A, B, and C [6]. Class A is highly infectious and causes massive epidemics within very short timespans, and includes plaque and cholera only. B-infectious diseases might cause epidemics and include acute respiratory syndrome (SARS), acquired immune deficiency syndrome (AIDS), viral hepatitis, poliomyelitis, highly pathogenic avian influenza, epidemic hemorrhagic fever, measles, epidemic encephalitis B, rabies, anthrax, bacillary and amebic dysentery, dengue fever, epidemic cerebrospinal meningitis, pertussis, diphtheria, neonatal tetanus, scarlet fever, brucellosis, gonorrhea, syphilis, leptospirosis, schistosomiasis, malaria, influenza A (H1N1), and typhoid fever. Class C involves less serious and fewer infectious diseases such as rubella, leprosy, mumps, leishmaniasis, and acute hemorrhagic conjunctivitis [7]. The new COVID-19 pandemic caused by SARS-CoV-2 has prevailed rapidly and infected around 17 million individuals worldwide (July 31, 2020) [8]. The basic reproduction or transmission number (R0 value) was estimated to be between 2 and 3.5, meaning that one patient can transfer the disease to 2 or 3 other individuals with a fatality rate of about 2 to 3%. SARS-CoV-2 caused many more deaths than its coronaviral relatives, while the mortality rates are considerably lower than MERS-CoV infections. The largest contributing factor towards enabling the control of MERS-CoV is the a low basic reproduction number (R0 around 1), indicating that each person transmits it to just one other individual (SARS-CoV R0 was around 4) [9]. The COVID-19 pandemic has posed an eminent threat to many countries. China took into consideration the surveillance role to control and manage epidemics. These rules include: Illustrating the natural history of pathogens, identifying case spread, initiating disease control measures, tracking infection epidemics, forecasting and preventing epidemics, and providing bases for strategy adaptation. Despite the measures, this epidemic caused health (Figure 1 and Figure 2) [10] and economic losses and led to isolation among the countries of the world and between peoples inside the same state. In response to that crisis, there is an urgent requirement to figure out different impacts of COVID-19 to public health.

Our review aims to evaluate strategies of the most affected countries from different continents all over the world (China, Italy, Germany, France, Spain, America, Canada, Brazil, UK, India, Japan, Singapore, Iran, Korea, and Australia) for confronting the epidemic as it explains the best practices that could help other countries to overcome current or any upcoming pandemic.

## 2. Public Health Responses to COVID-19

**Figure 3:**
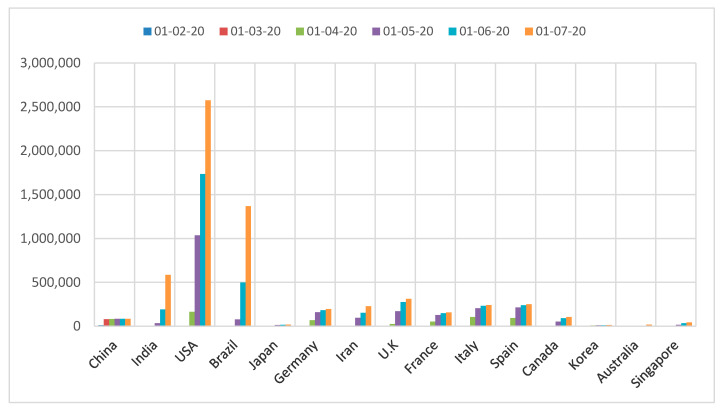
Change in numbers of confirmed cases over time [11,12,13,14,15,16].

Most countries were forced to announce emergency measures to protect vulnerable people and block ways of transmission due to the continuous increase in confirmed cases by time as reported in Figure 3 [11,12,13,14,15,16]. With regard to this escalating situation, governments have begun to develop strategies to resolve the pandemic cooperatively with international health agencies, i.e., Centers of Disease Control (CDC) and World Health Organization (WHO) that declared many precautions based on previous lessons from MERS and SARS diseases, as will be outlined in this section.

### 2.1. Chinese Response

The Chinese Lunar New Year holiday, which synchronized with the outbreak of COVID-19, is the most celebrative time of year in China. Usually, a large global migration takes place, as individuals travel back to their homes. Around five million people had left Wuhan [17]. Around a third of those people travelled outside the province of Hubei. Restricting people’s social contacts was critical to COVID-19 regulation. Key elements of such social distancing initiatives included that the Chinese government promoted people to stay home, dissuaded mass gathering, postponed or cancelled major events, and closed universities, factories, museums, libraries, schools, and governmental offices. Chinese people began to take steps to shield themselves from COVID-19, i.e., wearing protective masks, if they had to commute in public. Social distancing has been successful in limiting human to human transmission and cutting morbidity and mortality. More stringent steps are introduced such as isolation and quarantine. The Lunar New Year holiday was expanded by Chinese government. The holiday deadline was shifted to 10 March for Hubei province and 9 Feb for other provinces, so that the holiday duration was long enough to cover the alleged COVID-19 incubation time. Diagnosed people were segregated in hospitals. In Wuhan, in which a large number of infected people resided, people with mild or asymptomatic infections were quarantined at shelters called *Fang Cang* hospitals, which were public open areas, i.e., stadiums and convention centers that had been retooled for medical treatment. The Chinese government promoted and funded grassroots screening for contact tracking and early detection and encouraged hand-washing and surface sanitization. Home-based quarantine of people who were at the epicenters of epidemic and travelled to other places in China to curb the spread of virus to boarder populations. The government avoided panic amongst people by providing the updated information through media. Free medical care was introduced by the state to motivate patients to visit doctors as soon as possible and in good time to prevent further deterioration of the condition. The state guaranteed people’s daily needs [18].

### 2.2. Italian Response

The state with the second highest numbers of viral deaths worldwide. The government declared a state of emergency lockdown that began in Northern Italy and spread throughout the world. The fatality rate (7.2) was much higher than that of China (3.8). All Italian regions were known as “red zones” with extreme limits imposed on every public event. Italy responded to the situation with screening even for those without symptoms. Italy faced a persistent shortage of health care staff. The government announced a proposal to recruit 20,000 new doctors, nurses, and health workers to meet demand. Retired doctors and students who had finished their medical degree and are in the final year of specialist training, were called upon [19]. Coordinated intensive care units were equipped for COVID-19 positive patients. Continuous training for health care staff was crucial with applying dedicated protocols and full isolation [20]. The rules initially laid down approximately one month for schools’ closures and restrictions on people’s right to leave homes and two weeks for the suspension of business activities. The Italian government proposed an extension of lockdown steps [21]. To curb viral transmission, air travels were banned from China and Italian passengers were quarantined in China. Suspected cases were moved to pre-defined hospitals where the check for SARS-CoV-2 was available and infectious disease divisions were willing to isolate confirmed cases. Emergency Medical System of Milan Metropolitan area formed COVID-19 response team with main goal of resolving the viral pandemic without encumbering regular Emergency Medical System activities. The response team examined the health and clinical conditions of persons being screened to evaluate the need for hospitalization or home testing and subsequent isolation. This response team designed algorithm to identify COVID-19 suspected cases. The algorithm is continuously modified to comply with the regional directives [22].

### 2.3. German Response

In a speech about the Coronavirus pandemic, German Chancellor Angela Merkel approached the citizens directly. She explained the situation this way “It is serious. Take it seriously too!”. “Since World War II, there has been no other challenge to the country, where national solidarity was as important as right now”, she said. The German Chancellor announced stricter steps and declared 9 standards/rules for Germany. The main objective was to “reduce public life to the extent warranted”. This included restriction of the bare minimum connections, maintenance of a minimum distance to the public of at least 1.5 m, permission for people to go to work, doctors, shops, and play outdoor sports individually. However, gatherings in groups or meeting were no longer permitted [23].

### 2.4. French Response Plan

France, like other nations formed their Pandemic Influenza Plan (PIP) based on the recommendations for the contagion management by WHO. President Macron clarified that only collective national campaign can prevent the spread of infection, restrict deaths, and avoid the submergence of health service. French PIP aimed to alleviate pandemic by minimizing the number of civilian casualties and preserving machinations in particular economic activities. PIP included 4 stages: The 1st stage was to impede the introduction of outbreak to the world, 2nd stage to restrict viral growth and distribution in France, 3rd stage to attenuate the potential outbreak to minimum and 4th stage was returning to normalcy. First reported cases were Chinese nationals visiting France, so steps were rapidly taken to keep these cases in isolation. Contact tracing was held to identify people at risk of infection. The government cancelled all sporting events and schools were also closed. Authorities have repeatedly pronounced individual habits and requested protective masks for those who show signs of infections and for health workers so, public and private sectors were mustered to produce masks and disinfectants. To prevent viral transmission, France pressured the European Union to close the Schengen Treaty zone for all non-European citizens. Despite the strategy’s economic impacts, France scarified the entire society to combat COVID-19 [24].

### 2.5. Spanish Response

On 14 March, the Spanish government started the applications of safety measures, in order to flatten the curve 13 days after the exponential rate of virus start (R0 < 1); the day in which 20 new cases were registered for the first time. All people were forced to stay home through announcing the lockdown.

Spain has adopted some measures to control spread: Social distance, closure of most activities, e.g., cinemas, clubs and schools to avoid crowding [25]. Under supervision of the President of the Government, Pedro Sánchez, who described the crisis as: ”Unprecedented challenge”, “a global threat that recognizes no borders, colors or languages”, and an “extraordinary challenge that forces us to take exceptional measures”. He assured the importance of application of distance learning as much as possible to slow down viral spread. They reduced non-essential work to conserve support to different sectors including the vulnerable categories, the elderly, families with the lowest resources, and small business owners. Their strategy included increasing the awareness that each person in the community has a role in combating the virus; elderly people receive intensive care and the young follow the safety measures and social distancing. Everyone had to care of others and the sense of social responsibility was increased. Moreover, they had a continuously announced transparent data from the beginning beside their steps to prevent infection through following the guidelines and health monitoring protocol [26].

### 2.6. American Response

The Director-General of the World Health Organization (WHO) announced that the COVID-19 pandemic had triggered an international public health emergency. The United States department of Health and Human Service secretary announced on 31 January 2020 a U.S public health emergency, and the U.S. president legitimated a “Proclamation on Suspension of Entry as Immigrants and Non-Immigrants of Persons that Pose a Risk of Transmitting 2019 Novel Coronavirus”. This regulation restricts the entrance of American citizens and those with legal permanent residents and their families, especially those who have travelled to mainland China. The Centers of Disease Control and Prevention (CDC) and other governmental agencies, as well as state and local health centers, introduced proactive steps to limit COVID-19 propagation in the U.S. [27]. Such steps included the recognition of cases and their contacts, and the suitable care of travelers coming from China to the U.S. The correct actions were taken to (1) slow down virus spread; (2) prepare health care systems and encourage public willingness for pervasive transmission; and (3) clearly define infection and directly report to public health centers in order to make decisions and improve medical safeguards involving diagnosis, therapy, and vaccines [28]. Despite the fact that these initiatives were being enforced in anticipation of the virus in the U.S., the continued widespread dissemination of the virus was devastating. USA holds a negative record in regard to the pandemic, with the highest number of infections and deaths recorded worldwide.

### 2.7. Canadian Response

Public health and disease prevention programs in Canada were refashioned around guidelines and recommendations of Naylor and his group that were used before against SARS and entitled “Learning from SARS”. Experience with SARS affected positively Canada’s response to the COVID-19 outbreak. Most notably, correspondence concerning public health was greatly improved and digital media was progressed. There were some technological gaps like contrasting directives on the use of personal protective equipment but this has been mainly resolved. In airports, procurement were organized and rolling tests became faster [29].

### 2.8. Brazilian Response

Previous preparedness before incidence of infection was phenomenal in Brazil. On 22 January 2020, the health surveillance secretariat together with the ministry of health activated an emergency health operation center with low alerting level, which was raised later on 27 January when the first suspected corona virus case appeared. National Contingency Plan (NCP) for the COVID-19 and guidelines; based on information received from WHO were announced to be applied in all states. Quarantine law was imposed for protecting people. Isolation and exceptional restrictions on travelling was applied even before the appearance of the first case.

Currently, there is a rapid growth in cases in Brazil; 3904 cases and 114 deaths were registered only one month after the first confirmed case [30]. Trials to reduce cases were implemented and huge attention was paid towards availability of intensive care units (ICUs), diagnostic tests and ventilators needed for patients with COVID-19 [30]. Brazil suffered from political flounder, which constituted distraction in the middle of crisis. The government restricted the use of RT-PCR examinations to people with more severe symptoms leading to higher mortality rates. This was due to high cost of materials and shortage in qualified people and labs able to do the RT-PCR test and the needed transportation for samples to places, where tests are performed. Thus, people with mild symptoms or the asymptomatic caused the transmission of infection.

Dense populations on favelas made it impossible to follow the social distance. Moreover, illegal mining and logging in Amazon forests may have brought infections to remote areas. Scientific organizations, such as the Brazilian Academy of Sciences opposed Bolsonaro due to the decreased science budget, general security, and shortage of public services. Currently, there is increased production of personal protective equipment, ventilators, and diagnostic kits [31,32].

### 2.9. British Response

The United Kingdom (U.K.) government followed Health’s Department direct recommendations for travelling abroad with respiratory infections, especially travelling to Wuhan [33]. The U.K. National Health Service emphasized the importance of using personal protective equipment, obtaining a detailed history of travelling, and rapidly escalating suspicious cases with a dedication to isolate patients. Any confirmed cases of COVID-19 should be moved to an airborne high impact infectious disease center such as the two major centers in England (Royal Free Hospital in London and Newcastle Royal Victoria Infirmary). U.K. Chief Medical Officers told individuals who had toured Wuhan or Hubei Province over the past 14 days to stay at home and call National Health Service number 111. Such recommendations were also applied to people, who have visited Japan, Thailand, Hong Kong, Singapore, Taiwan, Macau, and Malaysia [34].

### 2.10. Indian Response

The world’s second most densely-populated country after China made the situation worse, since population density beside some other factors contributed to the wide viral transmission [35]. Poverty and money-related problems complicated combating strategies. If the government imposed social distance (1 m distance), many categories opposed the actions, especially craftsmen. Ignorance from Indians at first increased the number of infected people [36]. Then, the government imposed a strict lockdown for 55 days except for some services such as fire departments, police, and hospitals. Diagnostic kits were increased every day and in every state. Train coaches were turned to mobile wards for isolation. A phone application was launched called *Aarogya Setu* (Health Bridge) aiming to track people’s health [37]. Check points were built at borders to check people entering the country, and all borders were shut. The Ministry of Health and Family Welfare (MOHFW), India, increased awareness, took actions to control COVID-19 and guidelines on management; prevention and sample collection were announced. Also a hotline was created with a 24 h/7 days-a-week service to help people [38]. A huge budget of about US $2.1 billion was endowed for health sector to combat COVID-19. The Department of Science and Technology, Government of India tried to promote research in university institutes and started working in various directions to control the virus during the country’s lockdown. The Indian Council of Medical Research (ICMR) launched private labs with suitable safety regulations to test COVID-19 samples. ICMR reported that about 579,957 tests (as of 25 April 2020) were performed in India. Blood plasma therapy using the plasma of recovered patients with immunity against COVID-19 was applied to infected individuals. The Indian strategies paid the most attention for medical care requirements. Thus, the number of infected people is less than other countries due to exerted efforts by authorities to impose the strict lockdown. Yet even after lockdown removal (fully or partially) on 3 May 2020, the threats amplified [36]. On 9 June 2020, the Ministry of Health and Family Welfare (MoHFW) announced that 266,598 confirmed COVID-19 cases and 7471 deaths from 32 states especially the states of Maharashtra, Tamil Nadu, Delhi, and Gujarat. Hence, the case-fatality rate became 2.8% [39].

### 2.11. Japanese Response

It is not the first time for Japanese people to face a national crisis, as they previously experienced two atomic bombings in 1945, the sarin gas in 1995, and the H1N1 epidemic in 2009. Thus, fear and anxiety was dominating. Images, headlines, rumors and confirmation of human-to-human transmission in Nara Prefecture played a role. Anxiety-related behaviors appeared significantly in shortage of masks and sanitizers in drug stores, social rejection, discrimination against affected people [40]. However, preparedness and learning from previous lessons was effective. Japan reported low numbers of COVID-19-related deaths due to the following measures.

To prevent infection, emergency state was declared on 7 April 2020 and continued for a month. People were asked to stay home and stop un-essential activities. Japanese customs suited for social distancing, as they exclude handshaking, hugging, or kissing in greetings [41]. Usage of long-term care areas with the most vulnerable residents was temporarily suspended. Japanese people were asked to avoid crowded places with bad ventilation and conservation of physical distances according to recommendations of an expert committee [42]. Travels were restricted from and to Wuhan, and 565 Japanese citizens were asked to evacuate China. Subsequently, three flights transported them back home. Healthy individuals were isolated, prevented to move around and kept under medical observation at designated hotels, while others with disease symptoms upon arrival in Japan were admitted to hospitals [43].

### 2.12. Singapore Response

Singapore, the regional travel center in Southeast Asia, was one of the first places to be impacted by COVID-19. The Singapore strategies were based on back experience with SARS outbreak. An important lesson was to ensure cohesive response across all sectors, consistent leadership and guidance was crucial. Therefore, a multi-ministerial task force was established to provide central leadership for all government crisis management, before Singapore had its first COVID-19 incident. An intensified surveillance system was developed to monitor COVID-19 cases between hospital and primary care pneumonia patients. To promote this system, COVID-19 RT-PCR laboratory tests were rapidly expanded to all Singapore hospitals with 2200 tests per day for 5.7 million persons. Suspected and confirmed cases were isolated in hospitals immediately to avoid further transmission. Contact tracing was also started to determine their past locomotion before isolation to identify potential sources of infections. More than 800 Public Health Preparedness Clinics has been set up to facilitate the control of primary care of respiratory diseases. Incoming travelers were subjected to temperature and health checks at all airports and suspicious cases sent immediately to hospitals. Singapore’s community approach focused on social responsibilities while precautionary life kept going as usual. Social education was a key empowerment strategy and carried out through print, broadcast, and social media. Workers are empowered to continuously monitoring temperature and health and organizations are motivated to step forward their business plans. Schools remained opened with precautions. Even though these precautions were enforced, Singapore retained normality of daily life [44].

### 2.13. Iranian Response

By 5 March 2020, the viral spread increased, and all 31 provinces were affected. Then, by 3 April the number of confirmed cases reached 53,183 with deaths 3294 in Iran. The government prohibited many activities: Sale and export of face masks to legal entities were limited, commercial movements with China were prevented, and travel was banned. Cancelation of all public gatherings, including cinemas, concerts, theaters, postponement of weddings, parties, conferences, seminars, camps and collective sports, school closure, and establishing E- learning, reduced office hours for 2 h/day [45]. People were guided for hand-washing and wearing masks. Suspected and infected people with COVID-19 were isolated for 14 days [46].

Poor people were severely affected by quarantine; hence, the government financially supported them. The Supreme Council for Health and Food Security together with a special council for COVID-19 confessed essential deficiencies in policies regarding food security including delays in bills such as electricity, payment of bank loans. However, reductions in oil prices and oil selling due to sanctions significantly affected the ability of governmental support [47].

The Iranian Ministry of Health and Medical Education (MOHME) compiled the WHO guidelines for COVID-19 prevention and announced them through different platforms. Hotlines to answer questions and give advice on nutrition and mental health were available. National campaigns for increasing awareness and information were held to improve public knowledge. A website was launched (salamat.gov.ir) to help people and answer their questions [48]. The political situation in Iran impacted the economic infrastructure, which indirectly affected the health sector and the first-line defense against the virus. Thus, the burden scaled up. In addition, the weakness of the medical infrastructure, inadequate personal protective equipment and difficulties in importing them are all key factors. Quarantining cities was rather ineffective due to viral distribution throughout the country [49].

### 2.14. Korean Response

Korea’s infection alerting system has four levels: (1) Attention to the epidemic as the government began tracking, (2) caution if an epidemic reached the country and the government maintained a program of cooperation, (3) activation of response system that could be alerted regarding to spread of infection, and (4) development of a national response program, as the outbreak progressed and became serious. Four days after announcement of new cases in China, Korea began screening and enforced quarantine program at the airports. Everyone who had visited Wuhan during the past 14 days was asked to complete health questionnaire and to have 14 days of self-quarantine. If there was fever or respiratory ailments, they should call Korea CDC. Early recognition helped Korea remove the community infection and limit it to medical facilities which was an integral part of outbreak response. A 6-h rapid test was distributed in all health centers around the country. Korea CDC started recording the crisis to provide reliable data. Such reports included number and history of suspected cases with public guidance for prevention. Travel to China was cancelled. Korea goals were accomplished through 3 key strategies: 1st outbreak based on suppression and mitigation, 2nd risk awareness to encourage community involvement, and 3rd science-based and reality driven behavior [50].

### 2.15. Australian Response

**Figure 4:**
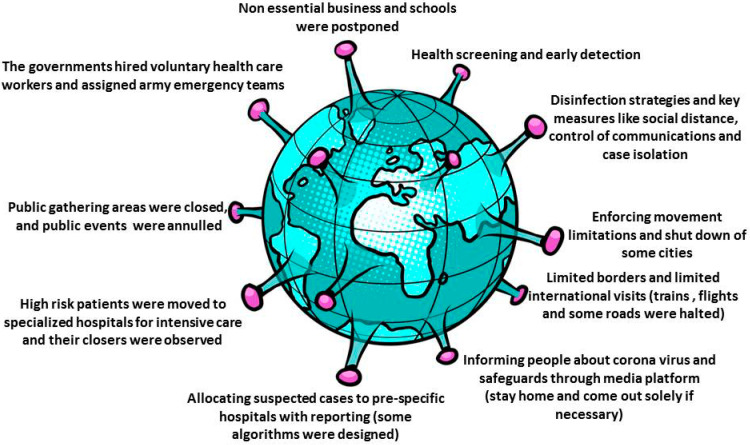
Common governmental responses to COVID-19.

Australia built its response to COVID-19 on the basis of its powerful healthcare system. Australia realized that people involved in primary care, elderly care, home care, and disability care need the same degree of support and safety as people working in hospitals in attempt to preserve both public and vital health care system to sustain the workplace of services. Good, coherent contact with the primary care staff and general public was very critical for the needed steps. Borders were shut down, non-essential facilities were closed, precautionary measures were in the places with infection risk, stringent social distancing were enforced together with quarantining of individuals with suspected infection or confirmed infection. The prime minister of Australia stated the implementation of the novel coronavirus emergency response program for Australian medical sector. The four strategic goals of the targeted plan were: protecting people from COVID-19 effects, maintain health care functional capacity, facilitate the most appropriate treatment of people with symptoms, manage, and control personal protective equipment. The Australian government introduced 2.4 billion primary care packages to safeguard all Australians. Primary care approach has main components: Telemedicine services, online infection control training provided to all caregivers, institution of general practice-led primary healthcare respiratory clinics (200 clinic) to transfer affected people away from other general practices [51]. Most governmental strategies are summarized in Figure 4.

Collectively, demographic diversity, standard of living of each country’s citizens, political state and health systems in addition to other factors led to various strategies being implemented across the globe trying to cope with the crisis. However, the collaboration and sharing of responsibility for controlling the pandemic through exchange of information between countries was the most important step. Taken together, countries facing COVID-19 or any other pandemic should consider control or closure periods and whether required or compulsory closure of unneeded workplaces and public entities as a first line of social distance measures can reduce transmission rate. The closure times should be adapted to the unique characteristics of the novel disease, i.e., the incubation duration and transmission routes, and the nature of these outbreaks. The main purpose of the pandemic control closure phase is to avoid the spread of disease by people with asymptomatic infections. Governments should use closure times to optimize effect, promotions, group screening, active communication, monitoring, isolation, and quarantine. Some countries have promoted their people’s consciousness across many channels, e.g., television, newspapers, and conferences. They have been resorting to the use of more modern health and education technologies i.e., E-learning and telemedicine to reduce the urge to go outside. Such a hybrid strategy is also backed up by analyses of responses to previous pandemics, which have shown that average attack rate reductions were more noticeable if social distance policies and other disease prevention steps were combined to prevent transmission.

## 3. Conclusions

SARS-Cov-2 spreads at an astonishing speed across the globe. On 30 January 2020, WHO announced the outbreak of COVID-19 an international public health emergency which impacted 77 countries (status: 4 March 2020) [52]. The speed and extent of pandemic detection, particularly early diagnosis and notification of new cases, is an important measure to monitor this infectious disease. Countries that have previous experience with viral infectious diseases (most commonly SARS), powerful primary care systems with helpful infrastructures, guidance rules and instructions, and community awareness with social responsibilities prove to be more effective in controlling the spread of infection and reducing its deleterious impacts. Numerous countries endeavor to construct an info-structure of national digital health in order to improve disease surveillance and link public health and clinical intelligence programs. Clear and open contact between governments and healthcare staff would be pivotal. It was the time for hospitals or agencies that engage in healthcare delivery to audit its protocols and consumables for all selected patients. Heads of State, global health leaders, private sector partners, and other stakeholders have accelerated global partnership to speed up the production of COVID-19 diagnostic and preventive tools. All governments should prepare the public for a second wave or another outbreak. National policy discussions about the future of the respective society should be initiated. COVID-19 is a tragedy for us all collectively, but it is also an opportunity to ask ourselves what kind of society we want after the pandemic fades away.
